# Generalizable stereo depth estimation with masked image modelling

**DOI:** 10.1049/htl2.12067

**Published:** 2023-12-23

**Authors:** Samyakh Tukra, Haozheng Xu, Chi Xu, Stamatia Giannarou

**Affiliations:** ^1^ Hamlyn Centre of Robotic Surgery, Department of Surgery and Cancer Imperial College London London UK; ^2^ Present address: Imperial College London Exhibition Rd, South Kensington Campus London UK

**Keywords:** computer vision, convolutional neural nets, learning (artificial intelligence), neural nets, stereo image processing

## Abstract

Generalizable and accurate stereo depth estimation is vital for 3D reconstruction, especially in surgery. Supervised learning methods obtain best performance however, limited ground truth data for surgical scenes limits generalizability. Self‐supervised methods don't need ground truth, but suffer from scale ambiguity and incorrect disparity prediction due to inconsistency of photometric loss. This work proposes a two‐phase training procedure that is generalizable and retains the high performance of supervised methods. It entails: (1) performing self‐supervised representation learning of left and right views via masked image modelling (MIM) to learn generalizable semantic stereo features (2) utilizing the MIM pre‐trained model to learn robust depth representation via supervised learning for disparity estimation on synthetic data only. To improve stereo representations learnt via MIM, perceptual loss terms are introduced, which improve the model's stereo representations learnt by explicitly encouraging the learning of higher scene‐level features. Qualitative and quantitative performance evaluation on surgical and natural scenes shows that the approach achieves sub‐millimetre accuracy and lowest errors respectively, setting a new state‐of‐the‐art. Despite not training on surgical nor natural scene data for disparity estimation.

## Graphical abstract

This research develops a novel stereo depth estimation method, integrating self‐supervised and supervised learning. It begins with masked image modelling for stereo‐semantic feature learning, then refines it through supervised training on synthetic data for disparity estimation. Enhanced by perceptual loss and model design, the method achieves sub‐millimeter accuracy in surgical and natural scenes, setting a new benchmark without requiring real‐world data.

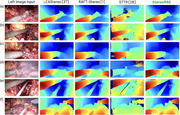

## 1 INTRODUCTION

Depth estimation is of paramount importance in minimally invasive surgery (MIS) facilitating, soft tissue 3D reconstruction, surgical robot navigation and augmented reality. A popular method of perceiving depth is with a stereo camera by estimating the horizontal displacement (disparity) from the left image pixels to the corresponding right. End‐to‐end deep learning stereo reconstruction methods have become state‐of‐the‐art. They are typically categorized as supervised and self‐supervised. Supervised learning methods directly predict disparity (regression) by training on ground truth disparity data [1, 2, 3, 4]. They usually undergo training using synthetic data [5], followed by fine‐tuning on real‐world scenes to overcome the limited availability of ground truth. Despite their popularity, they face several challenges in surgical applications. Discrepancy between training and inference data, limited ground truth data, lower image quality, restricted space, and a multitude of scene variations, are factors that lead to reduced performance.

Self‐supervised methods don't require ground truth disparity, making images‐only training data abundant. These methods optimize photometric re‐projection error through novel view synthesis [6, 7, 8, 9, 10]. Self‐supervised methods show potential, but are not as effective as supervised methods. This is because the photometric error can be optimized for a large range of disparity values, resulting in inconsistent geometry. Furthermore, the relative depth information between stereo images is inherently scale ambiguous. Making it challenging to learn robust representation of depth via self‐supervision, especially in scenes with occlusions, textureless regions, or repetitive structures, which is prevalent in surgery. Supervised learning is key for robust stereo depth, since training on ground truth provides a strong, unambiguous signal for learning accurate representations. A question emerges though; can we have the best of both worlds, i.e. unambiguous depth that is also generalizable to different scenes?

For achieving generalizability, visual representation learning is key. Learning robust feature representations also allows improved downstream performance as shown in [11, 12, 13]. These techniques utilize image reconstruction as a preliminary task, relying on the principle that by learning to predict patches in a masked image, useful representations about the context of the scene can be obtained for further tasks. This is proven by the enhanced data label efficiency on various standard benchmark datasets. Hence, in this work we experiment with this masked image modelling (MIM) approach, to train an encoder model to learn stereo feature representations, that can be fine‐tuned downstream to encode robust depth feature representations. This endows our model with both generalizable features via MIM and unambigous sharp depth via supervised‐learning for disparity estimation.

In this paper we build on [11] and propose StereoMAE a two‐stage training process which entails (1) training an encoder via MIM, to generate robust feature representations for left and right views, followed by (2) supervised training for disparity estimation. Furthermore, we enhance MIM in (1) by using perceptual similarity learning [14], leading to learned representations that effectively capture the intricacies of the scene and object boundaries without explicit guidance or manually designed inductive biases. Our contributions are the following:

- A novel approach for training stereo depth models by combining self‐supervised MIM and supervised stereo depth estimation. To the best of our knowledge, this is the first work to apply MIM for stereo depth estimation;
- We propose a new approach to boost MIM by incorporating perceptual similarity loss term for learning generalizable visual semantic concepts;
- We present a modular model architecture for combining any pre‐trained MIM encoder model with any off‐the‐shelf decoder to enhance depth estimation;
- Our joint MIM‐supervised approach enhances performance, yielding a generalizable model with sub‐millimetre accuracy in surgical depth estimation

Specific to the field of surgery the lack of datasets with ground truth, restricts state‐of‐the‐art stereo models from reaching sub‐mm precision on surgical scenes despite their generalizability. This is due to the need for deep understanding of scene semantics, a task beyond the capabilities of supervised learning alone. Hence, our method pairs MIM with StereoMAE model architecture, and perceptual learning to surmount the limited training data issue, achieving high generalizability and sub‐mm accuracy in surgery. This blend of generalizability and precision offers a significant advantage, expanding its potential use across diverse medical imaging tasks, not just specific surgical scenarios like laparoscopic surgery where depth estimation at sub‐millimetre accuracy is critical as it helps the localization and perception of surgical instruments, tumour and surrounding healthy tissue (essential in the development of autonomous task execution in robotic MIS). The performance of StereoMAE has been evaluated on (3) MIS datasets; SCARED [15], Hamlyn [16], and SERV‐CT [17], and on (2) non‐surgical scenes data; ETH3D [18] and Middlebury [19].

## 2 METHODS

The method is divided into two phases; (1) Pre‐training via MIM for enhanced representation learning followed by (2) supervised downstream fine‐tuning for stereo depth estimation. The learning framework for (1) is inspired by MAE [11]. Supervised training in (2) is based on RAFT‐Stereo [1]. However for (2) any training methodology from the supervised stereo depth literature can be used.

### 2.1 Stereo masked image modelling

MIM involves randomly masking pixels of an input image and training a model to predict the invisible content [11, 12, 13]. The intuition is that the model learns the representation of the masked pixels by inferring details from the surrounding valid pixels. In the end, if a model is capable of reconstructing the missing content with valid pixels, the model has learnt the context within the image by encoding its semantic features.

**FIGURE 1:**
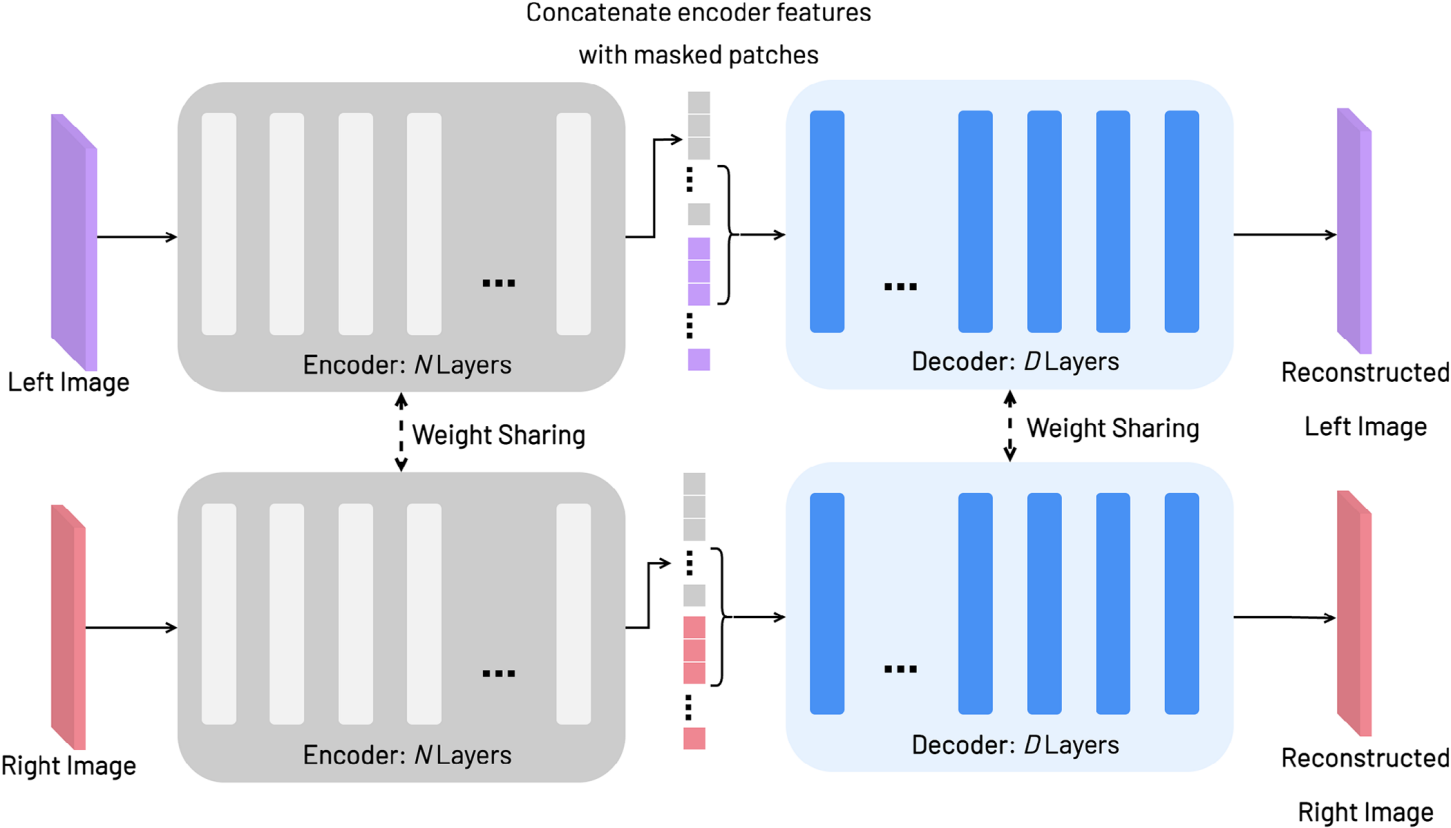
Masked image modelling (MIM) pipeline for StereoMAE comprising of a weight sharing transformer encoder–decoder model. Where N and D are the total number of transformer layers that make up the corresponding models.

We adapt the method in [11], which utilizes an auto‐encoder model for stereo images as shown in Figure 1. In particular, we construct the auto‐encoder model, using vision transformer (ViT) components [20]. The encoder consists of several layers of ViT‐Base (ViT‐B), a 12 layer transformer model. The custom decoder is composed of 8 transformer layers. Given the left image as input, it is first resized to 224×448, divided into patches of size 16×32 and 75% of them are randomly masked. The encoder is fed only the un‐masked patches as input, and it generates corresponding features representing the visible scene parts. These features are then concatenated with the patches that were not fed into the encoder and inputted into the decoder to reconstruct the full left image as output. The model is trained via pixel image reconstruction (photometric error). The same is done for the right input image. For the left and right input views, the weights of the encoder and decoder are shared, allowing them to learn joint global stereo representations for reconstructing the scene.

In our work, we do not train the aforementioned model using the mean square error (MSE) loss from the original work [11]. MSE, which compares pixel intensities, is a low‐level metric and insufficient for capturing complex structures in an image as it assumes pixel‐wise independence. Hence a model's capacity is wasted since high frequency global feature distribution will not be captured. Perceptual similarity, which mimics human visual perception [21], is a better metric for evaluating image similarity and capturing high‐level semantic features, essential for model generalization. Hence, we hypothesize, that training MIM with perceptual similarity will enhance the models' output quality and coherence. The focus is on learning high‐level relationships between elements in the image, not just low‐level details. Thereby reducing the model's vulnerability to masked regions and improving overall semantic content and structure capture.

Therefore, to train StereoMAE we utilize a perceptual loss for MIM for learning the required high‐level semantic features that represent the scene context (thereby improving its downstream generalizability). Inspired by [14] our perceptual loss comprises of three loss terms: (i) L1 (also known as absolute error loss, measures the absolute difference between the predicted and actual pixel values, calculated as the sum of the absolute differences between the two values). (ii) Feature matching (compares intermediate features from a pre‐trained model like VGG, to encourage learning similar features for the predicted and actual pixel values). Lastly, (iii) style transfer loss (computes correlations between feature maps extracted from a model like VGG, encouraging the predicted and actual image distributions match). L1 loss encourages sparse representations by optimizing absolute intensity values. The feature matching loss compares the feature representations of the reconstructed output and target image, while the style loss measures the difference in statistical distribution of high‐level feature activations, capturing image texture and aesthetics. To calculate (ii) and (iii) we utilize a pre‐trained feature extractor model, i.e. VGG16 (though any arbitrary model can be used).

(1) $$\begin{array}{c} L_{\text{mim}}^{I}=||G\left(I_{m}\right)-{I||}_{1}+\delta_{f}\sum_{j=1}^{J}\frac{1}{N_{j}}\left[|\right|\phi^{j}\left(G\left(I_{m}\right)\right)-\phi^{j}\left(I\right){\left|\right|}_{1}]\\ +\delta_{s}\sum_{j=1}^{J}\frac{1}{N_{j}}[||\Psi \left(\phi^{j}\left(G\left(I_{m}\right)\right)\right)-\Psi \left(\phi^{j}\left(I\right)\right){\left|\right|}_{1}] \end{array}$$

where, I is the original input image, $I_{m}$ is the masked input image, G is the StereoMAE model, ϕ is the feature extractor model and Ψ is the gram matrix. The loss weights $\delta_{f}$ and $\delta_{s}$ are 0.05 and 40.0, respectively. These weightings were selected via experimentation on our dataset (discussed in Section 2.3). The initial values were inspired by previous research in generative modelling [22, 23]. $L_{\text{mim}}$ is calculated for both the left and right views, making the full MIM loss for StereoMAE the following:

(2) $$L_{\text{mim}}=L_{\text{mim}}^{I_{L}}+L_{\text{mim}}^{I_{R}}$$

where, $I_{L}$ and $I_{R}$ are the left and right input views, respectively. MIM training was performed on a mixture of real natural and synthetic scenes (no surgical scenes were used).

### 2.2 Supervised downstream fine‐tuning

**FIGURE 2:**
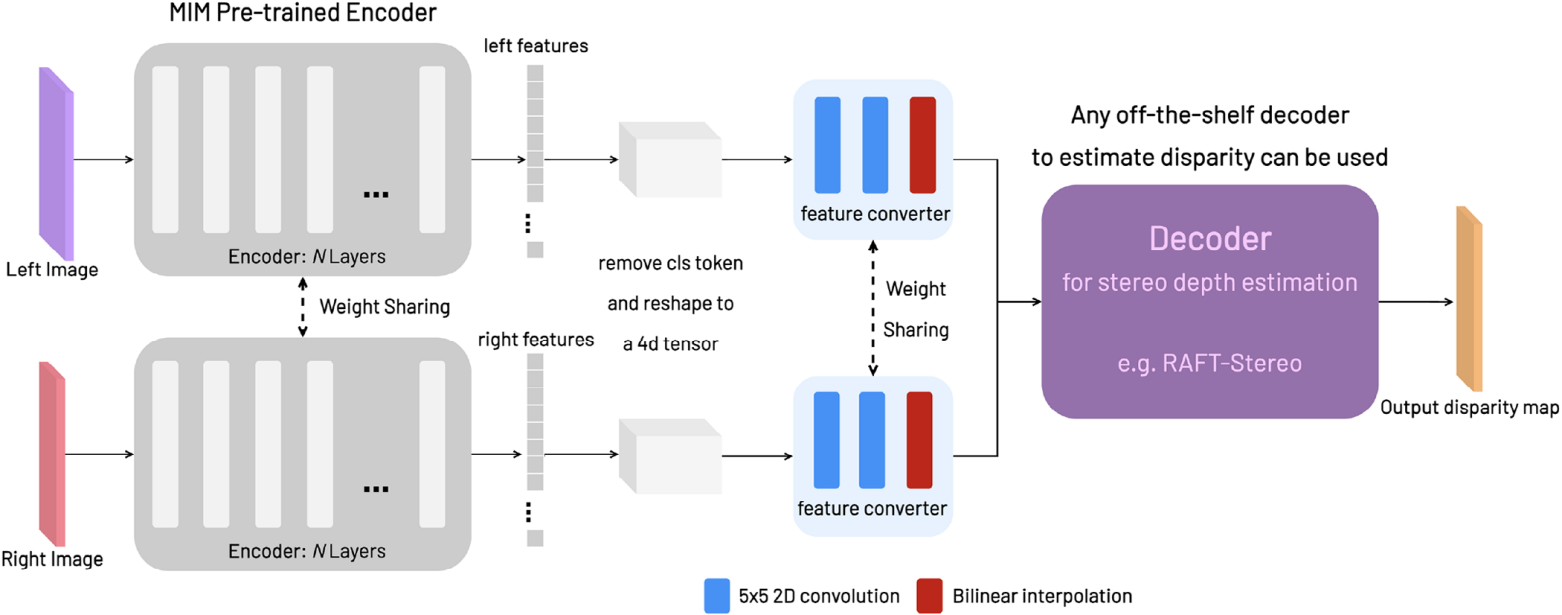
The downstream process of supervised stereo depth estimation training. Where the feature extractor encoders pre‐trained from MIM, are packaged with an off‐the‐shelf decoder for depth estimation. Any decoder of user's choice can be used.

Once the encoders have been pre‐trained via MIM in phase (1) we finetune them for disparity estimation. The full model architecture for downstream training is displayed in Figure 2. The architecture is designed to be modular such that the pre‐trained encoders can be combined with any off‐the‐shelf decoder of the user's choice. Thereby enabling a smooth transition from MIM to disparity estimation without further modifications. The pre‐trained ViT encoders generate features for the (un‐masked) left and right input images, and output a 3D tensor. The features have to be reshaped into a 4D tensor for it to be processed by the decoders which are typically composed of convolution layers. However, once reshaped, the positional encodings of the features change. To ensure the decoder focuses on the necessary elements of the feature map, we parse the feature map through a feature converter block; its purpose is to learn the transfer of MIM‐trained features to stereo‐disparity features and select the relevant elements needed for the decoder. It comprises of two 5×5 2D convolution layers and a bilinear interpolation module that resizes the tensor to a scale of choice. The scale depends on the type of decoder used. In our experiments we utilize the RAFT‐Stereo decoder [1]. The feature converter block essentially plays a crucial role in ensuring the appropriate transfer and transformation of features from the pre‐trained ViT encoder to the disparity decoder. In our experiments this is vital as the encoder is trained only for the MIM task, hence the features must be adapted for disparity downstream.

To train the final model we utilized a supervised‐learning loss comprised of the L1 error between the predicted and ground truth disparity. The RAFT‐Stereo decoder generates a sequence of predictions (from its multi‐level GRU networks), hence our L1 error is computed over the full sequence of predictions, [$d_{p}^{1},\text{…},d_{p}^{N}$], with exponentially increasing weights. If a different decoder that outputs a single disparity map is generated then, only the final single output $d_{p}$ would have been used for the loss calculation. Considering ground truth disparity is defined as $d_{\text{gt}}$, the loss is defined as

(3) $$L_{\text{sup}}=\sum_{i=1}^{Y}\gamma^{Y-i}\left|\right|d_{\text{gt}}-d_{p}^{i}{\left|\right|}_{1}$$

where, γ=0.9, is the weighting factor for the loss calculated for each scale and Y is the total number of scales. The supervised training for disparity estimation was only conducted on synthetic scenes (no surgical or natural scenes were used).

### 2.3 Implementation details

For MIM training, we use the ViT‐B architecture [11], trained for 150 epochs on a combination of the following datasets [24, 25, 26, 27, 28] (a total of 411,942 stereo image pairs). The input patch size is fixed to 16×32 and we mask 75% of input patches during training. The ViT‐B encoder, comprises of 12 transformer layers (N), each with 12 self‐attention heads and the final hidden dimension output is of size 768. The decoder architecture comprises of 8 transformer layers (D) each with 16 self‐attention heads and the hidden dimension output of size 512. The data augmentation strategies include (1) resizing / cropping image to 224×448, (2) adding random distracting shapes like rectangles of varying size and (3) random colour jittering. We train with a batch size of 8 on 4‐Nvidia Tesla GPUs. Adam optimizer with a learning rate of 0.00015, and weight decay of 0.05 (cosine strategy), 40 warm‐up epochs and the momentum parameters $\beta_{1}$ and $\beta_{2}$ are 0.9 and 0.95 respectively were used. For downstream training, the pretrained ViT‐B model is used as feature extractor with the RAFT‐stereo decoder architecture, for disparity estimation. The same training strategy as [1] was utilized, though since our model is modular, it can be combined with any downstream stereo‐decoder model and training method. For the downstream training of disparity estimation, only the Sceneflow training split [25] was used (a combination of FlyingThings, Monkaa and Driving). Hence, only synthetic data was used for training. Note, synthetic data is used for perfect, unambiguous ground truth disparity. Synthetic surgical scenes can also be used for training, provided high quality disparity maps are available. Once, trained inference operates at 14 frames per second (fps) on a single Nvidia Tesla GPU (where frames here includes both left and right pairs).

## 3 EXPERIMENTAL RESULTS AND DISCUSSION

**FIGURE 3:**
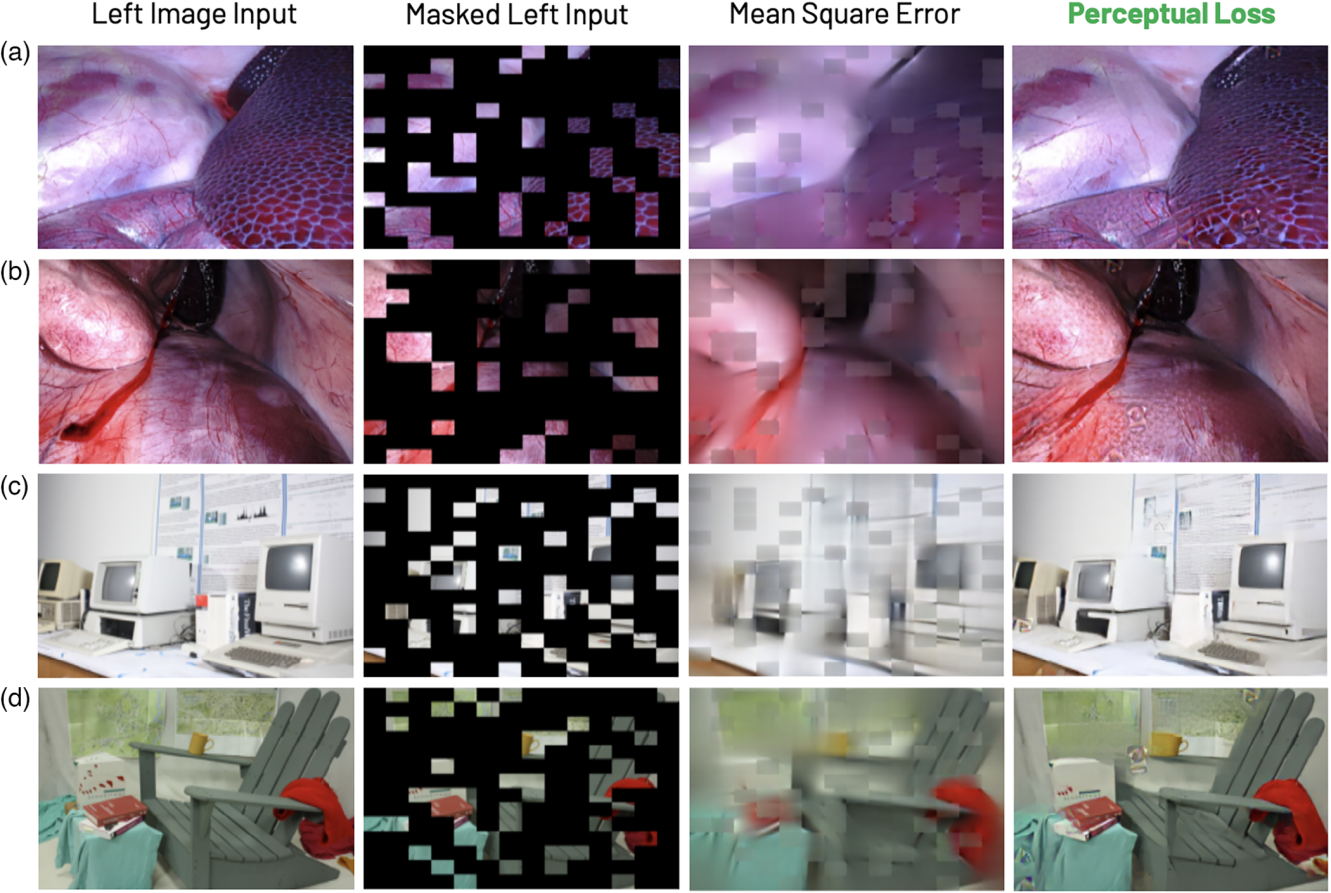
Reconstructions of the left images on (a, b) SCARED and (c, d) Middlebury samples. Results of MIM pre‐trained StereoMAE via mean square error loss [11] and the proposed perceptual loss shown in 3rd and 4th columns, respectively. The inputs were masked at 75% mask‐to‐image ratio. StereoMAE was not trained on these datasets.

**FIGURE 4:**
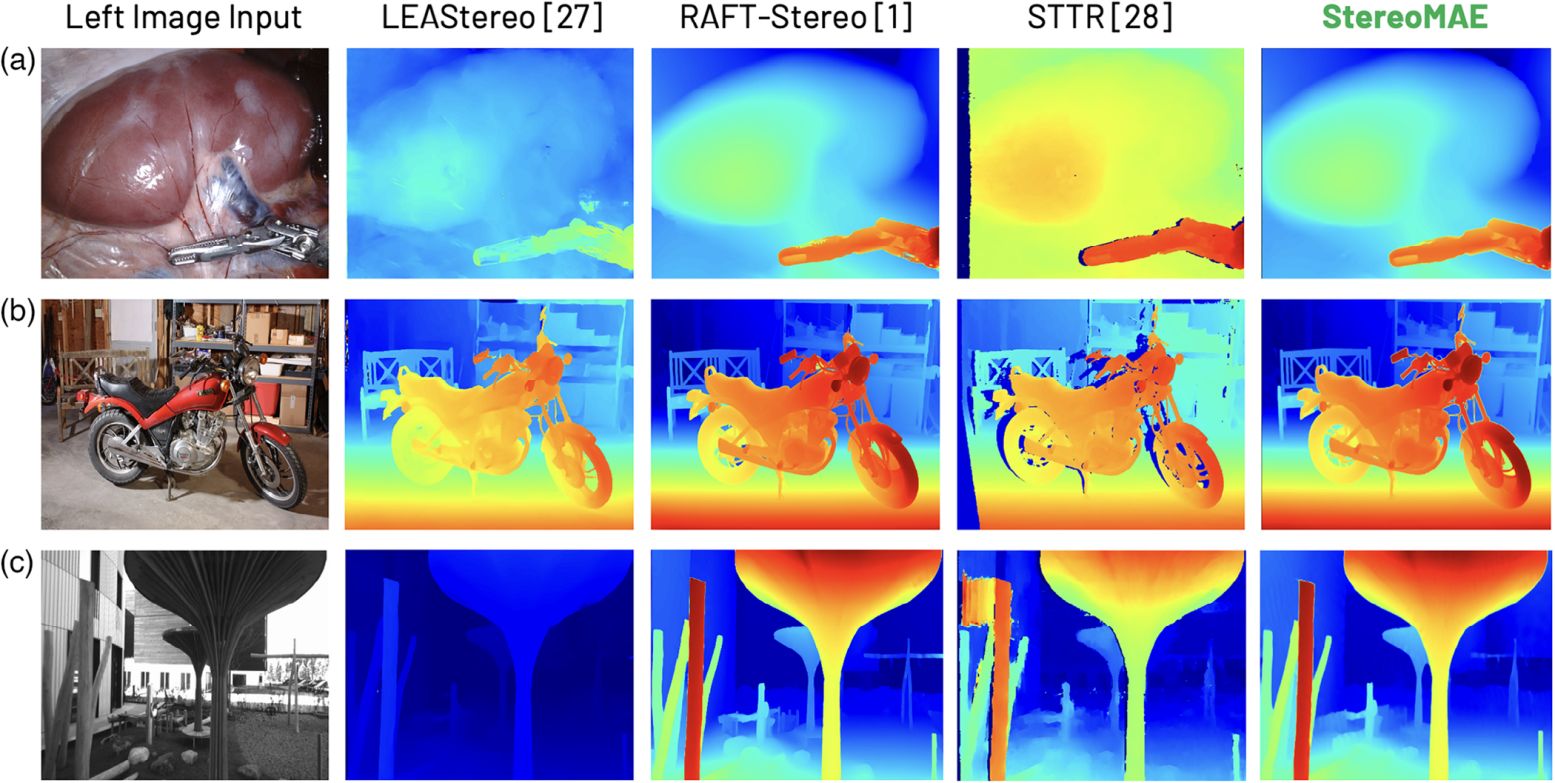
Left disparity outputs on (a) SCARED, (b) Middlebury, and (c) ETH3D samples. All models were trained only on synthetic data.

**FIGURE 5:**
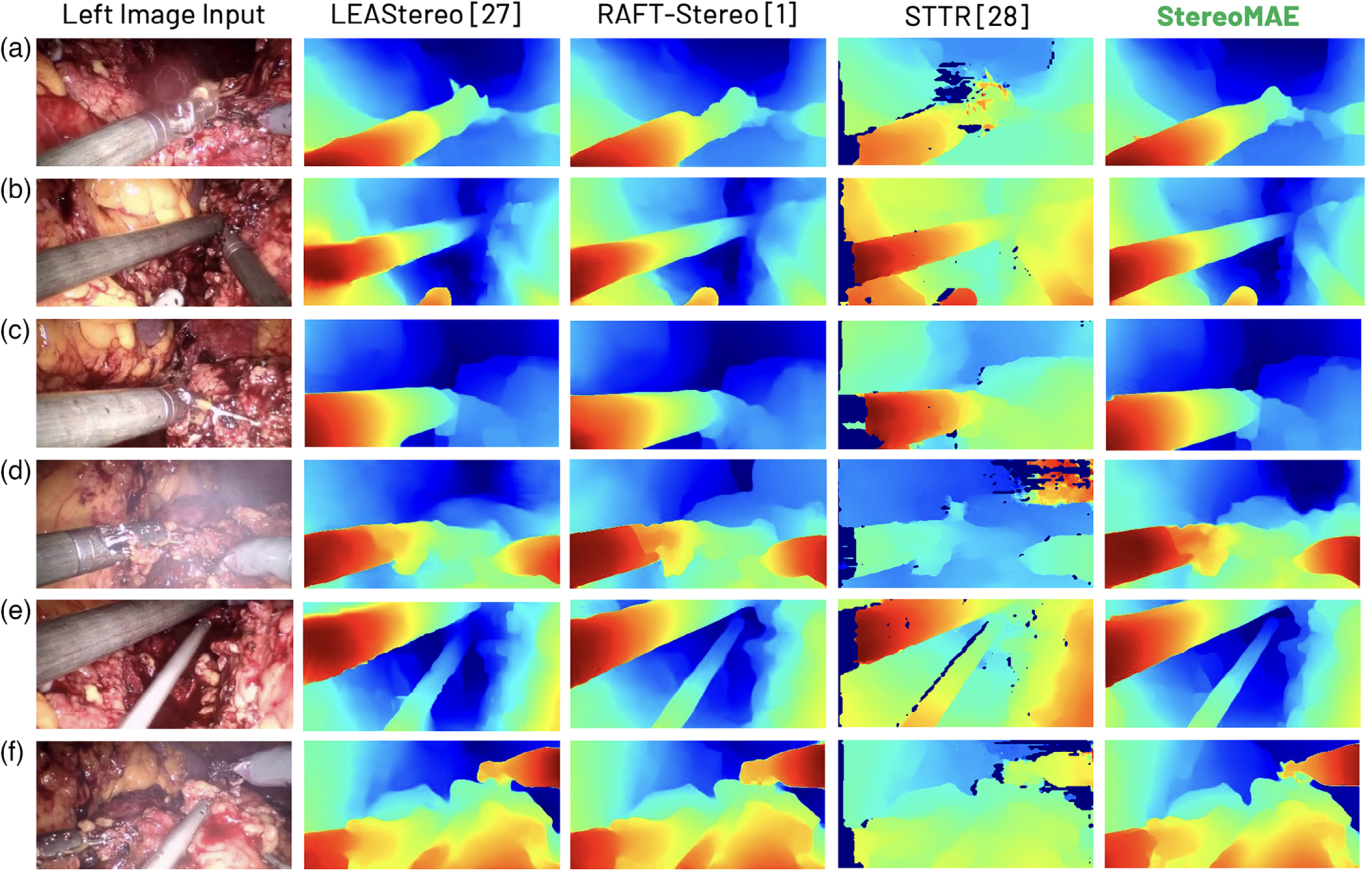
Left disparity outputs on the Hamlyn MIS data on different (a–f) samples. All models were trained only on synthetic data.

We evaluated the performance of our model on both surgical and natural scenes, despite not training on them. The following datasets were used for testing; SCARED, Hamlyn, SERV‐CT, ETH3D, and Middlebury datasets. To compare our results with other methods in the literature, we calculate the standard metrics for stereo disparity evaluation, i.e. average end‐point‐error (EPE) and the percent of pixels with EPE greater than a specified threshold (0.5, 1.0, and 2.0). Specifically for SCARED and SERV‐CT, we also calculate the mean absolute depth error in mm on test sets 1 and 2. We also exhibit qualitative performance of our MIM pre‐training and downstream disparity estimation in Figures 3 and 4. Furthermore, due to lack of ground truth available in Hamlyn MIS data we show qualitative downstream performance comparison with other SOTA methods in Figure 5.

Figure 3 compares the reconstructed outputs of StereoMAE trained via the MSE loss from [11] and the proposed perceptual loss, on unseen datasets. It can be observed that StereoMAE trained with perceptual loss outperforms MSE in all scenes. When using perceptual loss, we observe significant increase in the fidelity of the reconstructed patches with finer textural and structural details. Exhibiting perceptual loss aids the model in learning higher‐level feature representations that can generalize to any scene type despite never having observed surgical scenes. When finetuned for disparity estimation, StereoMAE visibly generates sharper depth maps despite only being trained on synthetic data, as shown in Figure 4. Specifically, in sample A, StereoMAE generates the fine details on the robotic tool, whereas other methods either fail to achieve the correct disparity range or miss fine details around the edges. Similarly in B and C, StereoMAE generates less holes around the edges, as can be seen in the wheels/handle of the bike in B and the structures in the background in C. Hence, by learning a generalizable feature distribution for stereo image representations and disparity estimation, without any training on datasets from Figure 4, StereoMAE creates smooth yet fine disparities. We also conducted an ablation study by creating a new StereoMAE variant. It pairs the StereoMAE encoder with HITNet's decoder [4], fine‐tuned on the Scene Flow dataset. It outperforms the original HITNet model in ETH3D, Middlebury and SCARED evaluations, confirming our method's decoder‐independence and effectiveness in enhancing any decoder for disparity estimation. Original HITNet achieves EPE of 0.31, 1.62 on ETH3D and Middlebury, respectively and 4.12 mm depth error on SCARED. Whereas our variant achieves 0.25, 1.27, and 2.98 mm error, respectively. Our approach, as demonstrated in Figure 4, is independent of baseline, lighting, image type (like ETH3D's greyscale), or specific scene types. Despite training solely on simulations, it attains sub‐mm accuracy (shown in Tables 2 and 3) on surgical scenes, delivering SOTA performance on simulated, natural, and surgical scenes alike. Affirming the significance of learning deep semantic features via MIM in pre‐training encoders. This is further reflected in Figure 5 where it is clear samples A, B, and E StereoMAE generates sharper details around the background anatomical structures and the edges of the robotic tools. Furthermore StereoMAE in sample D resolves disparity for the anatomical structures and the left‐robotic tool even in the presence of smoke. Despite not training on surgical scenes, StereoMAE consistently outperformes competitors by generating sharper disparity maps under challenging lighting conditions and image resolution.

The quantitative evaluation in Tables 1, 2, 3 also show our model outperforming previous state‐of‐the‐art stereo depth estimation models in Stereo‐benchmark datasets. StereoMAE achieves lower EPE across the board on all datasets, despite not being trained on them. In Table 1, SCARED benchmark only shows EPE and 3px error as they are the most commonly reported by previous methods on this dataset [30]. Furthermore, as shown in Tables 2 and 3, StereoMAE also sets a new record on surgical scenes, achieving sub‐millimetre accuracy in depth estimation. This exhibits the importance of visual representation learning for developing generalizable feature distributions, and still retaining the fine details of disparity estimation from supervised learning. Enabling generalizability to surgical images, without training on them.

**TABLE 1:** Results of generalization without fine‐tuning on ETH3D, Middlebury, and SCARED datasets. Lower is better for all metrics. Method highlighted in bold is best.

	ETH3D				Middlebury 2014 (H)				SCARED Small	
	EPE	0.5 px	1 px	2 px	EPE	0.5 px	1 px	2 px	EPE	3 px
Method	(pixel)	(%)	(%)	(%)	(pixel)	(%)	(%)	(%)	(pixel)	(%)
LEAStereo [29]	0.63	22.50	8.54	3.94	3.20	49.44	28.51	16.03	22.62	27.07
STTR [30]	1.29	30.37	11.33	4.85	2.22	40.30	22.00	12.21	6.03	9.52
RAFT‐Stereo [1]	0.27	8.10	2.33	0.96	1.12	24.28	14.02	8.43	1.16	4.59
StereoMAE	**0.22**	**6.77**	**2.10**	**0.78**	**0.98**	**22.14**	**11.33**	**7.39**	**0.96**	**3.94**

**TABLE 2:** Mean absolute depth error (mm) on SCARED. Best results are highlighted in bold. None of the models were fine‐tuned on SCARED, inference only.

	SCARED 2019		SCARED Small
	Testdata 1	Testdata 2	Small 19 [30]
Methods	MAE (mm)	MAE (mm)	MAE (mm)
LEAStereo [29]	3.82	4.51	1.48
STTR [30]	4.14	5.91	11.31
RAFT‐Stereo [1]	3.74	4.28	1.01
StereoMAE	**1.66**	**2.01**	**0.92**

**TABLE 3:** Mean absolute depth error (mm) and EPE on SERV‐CT data. Best results are highlighted in bold. None of the models were fine‐tuned on SERV‐CT, inference only.

	SERV‐CT			
	Experiment 1		Experiment 2	
Methods	MAE (mm)	EPE	MAE (mm)	EPE
LEAStereo [29]	3.14	3.05	3.66	5.39
STTR [30]	16.60	7.54	25.69	14.52
RAFT‐Stereo [1]	2.03	1.32	2.61	2.27
StereoMAE	**0.99**	**1.06**	**1.19**	**1.68**

## 4 CONCLUSIONS

Accurate and generalizable stereo depth estimation is crucial for surgical applications. Supervised learning is effective but limited by the scarcity of ground truth data in surgical settings, restricting their generalizability. Self‐supervised methods have issues with scale ambiguity and inaccurate disparity prediction. Our StereoMAE approach demonstrates that by integrating MIM for feature representation learning with supervised depth estimation, achieves the benefits of both methods. Despite training only on synthetic data, StereoMAE shows generalizability to surgical and natural scenes, achieving sub‐millimetre accuracy in surgical scenes. This is attributed to robust feature representations learned during MIM pre‐training, which enhances the model's performance for subsequent tasks. Future work will explore alternative designs and loss terms to enhance MIM and improve stereo depth estimation.

## NOMENCLATURE

StereoMAE: Stereo masked autoencoder
MIM: Masked image modelling
MIS: Minimally invasive surgery

## AUTHOR CONTRIBUTIONS

All authors contributed to the conceptualization and design of the study. Samyakh Tukra wrote the basline script for training StereoMAE both pre‐training and downstream finetuning. Haozheng Xu and Chi Xu performed the analysis, Haozheng Xu further aided with the interpretation of the results. All authors contributed to manuscript revision, and read and approved the submitted version.

## CONFLICT OF INTEREST STATEMENT

The authors declare no conflicts of interest.

## Data Availability

All data used in the study is open sourced. Hence, no data are available.
